# Supplementary material for: Machine Learning Algorithms Applied to Identify Microbial Species by Their Motility
Source: Life (Basel). 2021 Jan 12;11(1):44. doi: 10.3390/life11010044 (PMC7828299; doi:10.3390/life11010044)
Supplement: Supplementary file 1 [file life-11-00044-s001.pdf]

life

Supporting Information for

Machine Learning Algorithms Applied to Identify Microbial Species by Their Motility

Max Riekeles [1,*], Janosch Schirmack [1] and Dirk Schulze-Makuch [1–4]

[1] Astrobiology Group, Center of Astronomy and Astrophysics, Technical University Berlin, , Berlin 10623, Germany; j.schirmack@tu-berlin.de (J.S.); dirksm@wsu.edu (D.S.-M.)
[2] GFZ German Center for Geosciences, Section Geomicrobiology, 14473 Potsdam, Germany
[3] Leibniz-Institute of Freshwater Ecology and Inland Fisheries (IGB), Department of Experimental Limnology, 16775 Stechlin, Germany
[4] School of the Environment, Washington State University, Pullman, WA 99164, USA
* Correspondence: riekeles@tu-berlin.de

Contents of this file

Text S1
Figures S1 and S2
Table S1

Additional Supporting Information (Files uploaded separately)

Captions for Datasets S1 to S14

Introduction

Here, we provide an extension of the information, given in the article, including an overview about the classification algorithms used. In this context, we also refer to the Python-codes used and refer to the Matlab files for the calculation of the aggregated features, as well as the simulation of Brownian motion.

Text S1

The simulation of Brownian motion, which we compared to the microbial motility and used for the classification algorithms, assumed 50 particles with a diameter of 0.5 µm, 50 particles with a diameter of 2 µm, and 100 particles with a diameter of 1 µm. These simulated 200 particles were compared to 200 real microbial pathways (50 of *E. coli*, 50 of *P. haloplanktis*, 50 of *P. halocryophilus,* and 50 of *B.* subtilis, see data set 4 for the motility statistics of the particles and data set 11 for the simulation of Brownian motion.)
"In Figure S1, we show the mean displacement of particles of similar size due to Brownian motion over time at 4°C and 25°C, compared with the observed microbes. We see that the microbes have a much higher displacement than the simulated particles due to Brownian motion. The temperature change has affected the microbial species differently (see main text, Figure 3a), but causes a relatively small change in Brownian motion.
The distribution of displacement due to Brownian motion is a Gaussian distribution. Both, the tumbling of the run-and-tumble motion, and the flick of the run-reverse-flick motion of bacteria, randomizes the swim direction and approximates a Gaussian distribution as time goes by. In Figure S2, we show the histograms of the mean speeds of the species on the left and the corresponding Q-Q plots on the right. The alignment of the dashed line represents for each case a perfect Gaussian distribution. We see for *E. coli* that the distribution tends to be distributed somewhat uniformly. We see for *B. subtilis* a distribution that is dominated by its strong outliers. The two species *P. halocryophilus* and *P. haloplanktis*, seem to follow fairly well a Gaussian distribution."

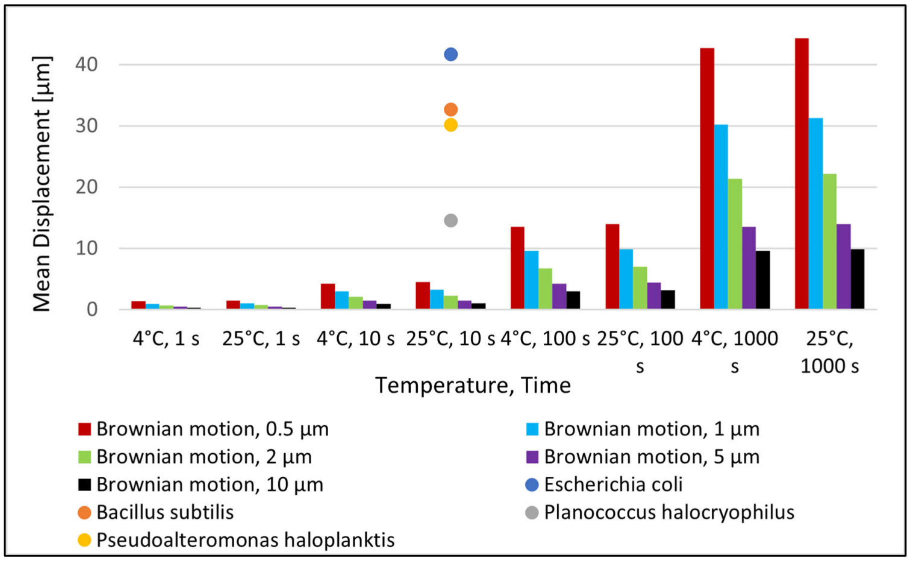

Figure S1. Mean displacement with respect to time.

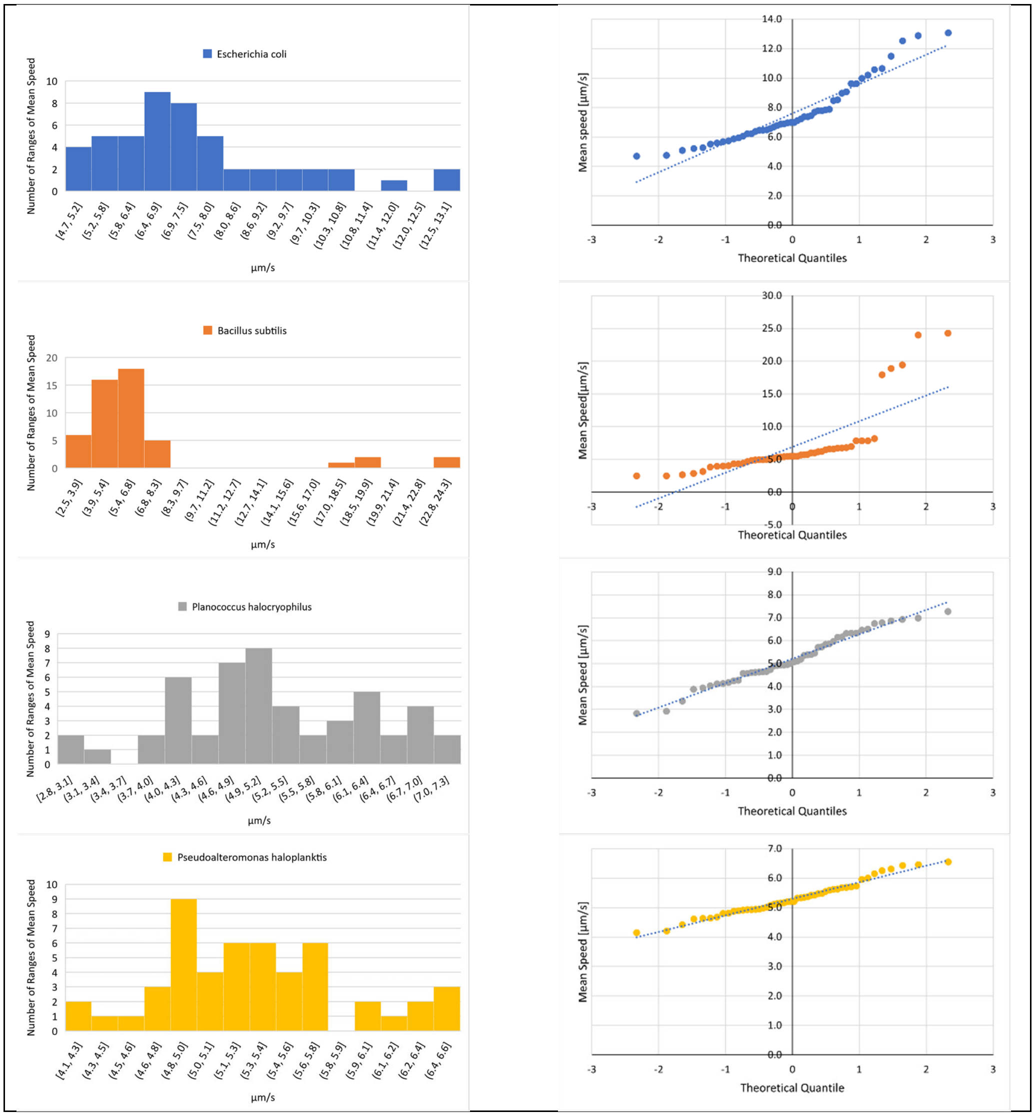

Figure S2. Mean speed distribution. a (first row): histogram (left) and Q-Q plot (right) for *E. coli.* b (second row): histogram (left) and Q-Q plot (right) for *B. subtilis.* c (third row): histogram (left) and Q-Q plot (right) for *P. halocryophilus.* d (bottom row): histogram (left) and Q-Q plot (right) for *P. haloplanktis.*

Table S1. Used Classifier (for more information on the used classifiers see James et al. (2013).

Algorithm	Description
Logistic Regression Classifiers (LRC)	This algorithm assumes a linear relationship between the input variables and the output variables. The coefficients of logistic regression are determined by a maximum-likelihood estimation. Since the response variable is categorical, it is used to solve classification problems.
Linear Discriminant Analysis (LDA)	This algorithm reduces the dimensions and maximizes the separability among known categories. It is a method used to find a linear combination of features that characterizes or separates classes of objects. When the model is trained, the parameters of the Gaussian distribution of each class are found. The distribution parameters are used to calculate boundaries, which determine the class of new data. When the classes are well separated, the parameter estimates for the logistic regression model are surprisingly unstable. LDA does not suffer from this problem.
K-Nearest Neighbor Classifiers (KNN)	These classifiers approximate the function only locally and all computation is deferred until function evaluation. The classifier first identifies the K points in the training set closest to the observed variable. The K neighbors are taken from a set of objects for which the class is known. This is the training step of the algorithm. The choice of K has a drastic effect on the KNN classifier, where small numbers of K lead to overly flexible results, and too high numbers of K lead to static results.
Classification and Regression Trees (CART)	A tree classifier consists of branching conditions, where the value of a predictor is compared to a trained weight. The number of branches and the number of the weights are determined in the training process. Either the Gini index or the entropy are usually used to evaluate the performance of a particular branch split, when building a tree. This kind of algorithm is easy to interpret and fast to fit and needs low memory usage.
Naïve Bayes Classifier	This classifier assumes that the presence of a particular feature in a class is unrelated to the presence of any other feature. It classifies new data based on the highest probability of its belonging to a particular class. It is best used for a small dataset containing many parameters.
Support Vector Machines (SVM)	Classifies data by finding the linear decision boundary that separates all data points of one class from those of the other class. The SVM chooses the hyperplane by maximizing the margin between classes, when the data is linearly separable. If the data is not linearly separable, a loss function is used to penalize points on the wrong side of the hyperplane. Often a kernel transform is used to transform nonlinearly separable data into higher dimensions, where a decision boundary can be found. When the classes are well separated, SVMs tend to perform better than logistic regression, when the classes are more overlapping, logistic regression is often better.

Data Set S1. *ds01*: Motility Data of the four species. Note: “Distance” in μm, “Velocity” in μm/s, “Angle” in Degree.

Data Set S2. *ds02*: Aggregated motility data over a period of ten seconds for the four species. Note: “Distance” in Pixel (1 Pixel equals 0.11 μm).

Data Set S3. *ds03*: Motility data for the microbes for Figure 1.

Data Set S4. *ds04*: Aggregated Motility data of microbes and aggregated motility data of simulated biotic movements. Used for the automated classification biotic vs abiotic movements.

Data Set S5. *ds05*: Python code of the KNN Cassifier. Compiled with Python 3.7.

Data Set S6. *ds06*: Python code of the CART Classifier. Compiled with Python 3.7.

Data Set S7. *ds07*: Python code of the LDA Classifier. Compiled with Python 3.7.

Data Set S8. *ds08*: Python code of the LRC Classifier. Compiled with Python 3.7.

Data Set S9. *ds09*: Python code of the NB Classifier. Compiled with Python 3.7.

Data Set S11. *ds11*: Matlab file for the creation of movement due to Brownian motion and of its aggregated motility information. Compiled with Matlab R2019b.

Data Set S12. *ds12*: Matlab file for the calculation of the motility information of the X/Y-information of the particle observations. Deployment of the aggregated motility information. Compiled with Matlab R2019b.

Data Set S13. *ds13:* Detailed Information of classification results “biotic vs abiotic”. All classifiers, all feature combinations. Note feature names: Mean Speed= Mean Speed; Sd= Standard Deviation Speed; Ra= Relative amount of clockwise directional change; La= Relative amount of counterclockwise directional change; Za= Relative amount of low directional change; Aa = Average directional angle; Sda= Standard deviation of directional changing angles; Abstand= Mean Distance of Particles after ten seconds; SmallSpeed = Relative amount of low speed.

Data Set S14. *ds14:* Detailed Information of species classification results”. All classifiers, all feature combinations. Note feature names: Mean Speed= Mean Speed; Sd= Standard Deviation Speed; Ra= Relative amount of clockwise directional change; La= Relative amount of counterclockwise directional change; Za= Relative amount of low directional change; Aa = Average directional angle; Sda= Standard deviation of directional changing angles; Abstand= Mean Distance of Particles after ten seconds; SmallSpeed = Relative amount of low speed.
